# A Case of Varicella Zoster Virus Meningoencephalitis With Generalized Chickenpox-Like Rash

**DOI:** 10.7759/cureus.77178

**Published:** 2025-01-09

**Authors:** Tomoko Yamaguchi, Mariko Okada, Takuya Fukuoka, Yoshihiko Nakazato, Toshimasa Yamamoto

**Affiliations:** 1 Department of Neurology, Saitama Medical University, Saitama, JPN

**Keywords:** chickenpox-like rash, dementia, disseminated zoster, meningoencephalitis, varicella zoster virus (vzv)

## Abstract

We report the case of a 91-year-old man with varicella zoster virus (VZV) meningoencephalitis who presented with acute progressive delirium. Brain MRI revealed multiple small vessel lesions, and cerebrospinal fluid testing came back positive for VZV DNA by polymerase chain reaction. We assume that VZV had reactivated since he was elderly and had lung cancer. The fact that there were no skin eruptions in the early stage made the diagnosis difficult. If a patient without skin rashes presents with acute progressive cognition, VZV should also be listed in the differential diagnosis.

## Introduction

In immunocompromised patients with varicella zoster virus (VZV) infection, meningoencephalitis may develop from viremia. It has been reported that in viremia, the virus directly damages the walls of blood vessels in the central nervous system, causing encephalitis and myelitis due to vasculitis. Vasculitis caused by VZV is prone to vascular disorders such as cerebral infarction when relatively large blood vessels are affected, and demyelinating lesions are likely to occur in small vessel types [1]. VZV infection sometimes does not show skin eruptions in the early stages, which can make it difficult to diagnose VZV meningoencephalitis. Herein, we report a case of VZV meningoencephalitis in an elderly patient with rapidly advanced delirium without skin eruptions in the early stages.

## Case presentation

**Figure 1:**
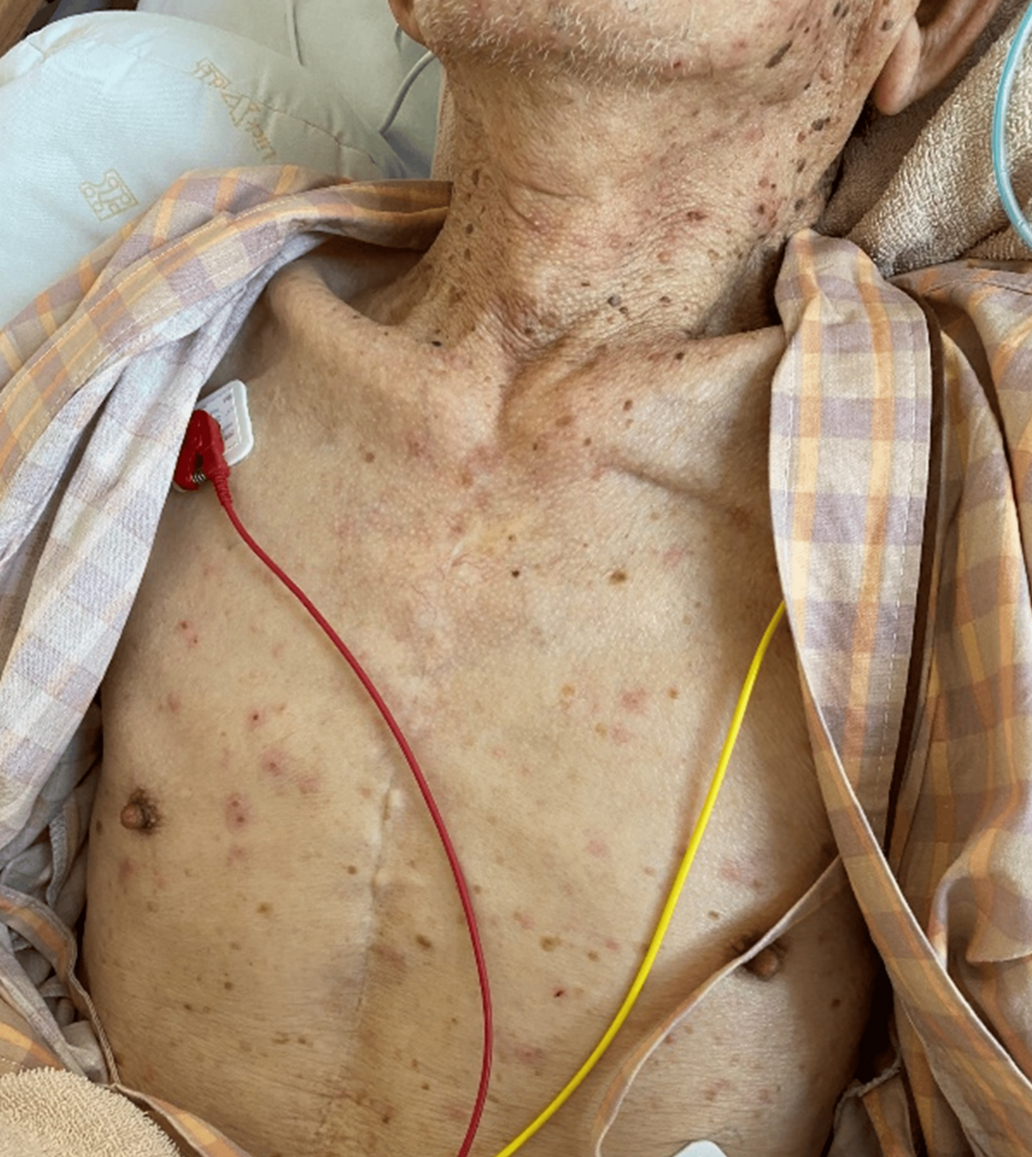
Physical examinations showing skin eruptions on the entire body surface.

A 91-year-old man arrived at the emergency department with progressive confusion over the past four days. Three days before admission, abnormal behaviors such as fiddling with things in the toilet and sprinkling detergent on the floor were observed. The following day, he was diagnosed with Alzheimer's disease at another hospital. At that time, he had no skin eruptions or fever. Although he had a past history of coronary artery bypass grafting, chronic heart failure, and a brain aneurysm, cognitive decline was not noted previously. On admission, his blood pressure was 220/110 mmHg, pulse rate was 90 beats/min, and body temperature was 38.1℃. Physical and neurological examinations revealed skin eruptions on the entire body surface and disturbance of consciousness. Meningeal irritation signs were positive. VZV infection was suspected because of a generalized chickenpox-like rash, and the VZV antigen test of the vesicle was positive (Figure 1).

**Figure 2:**
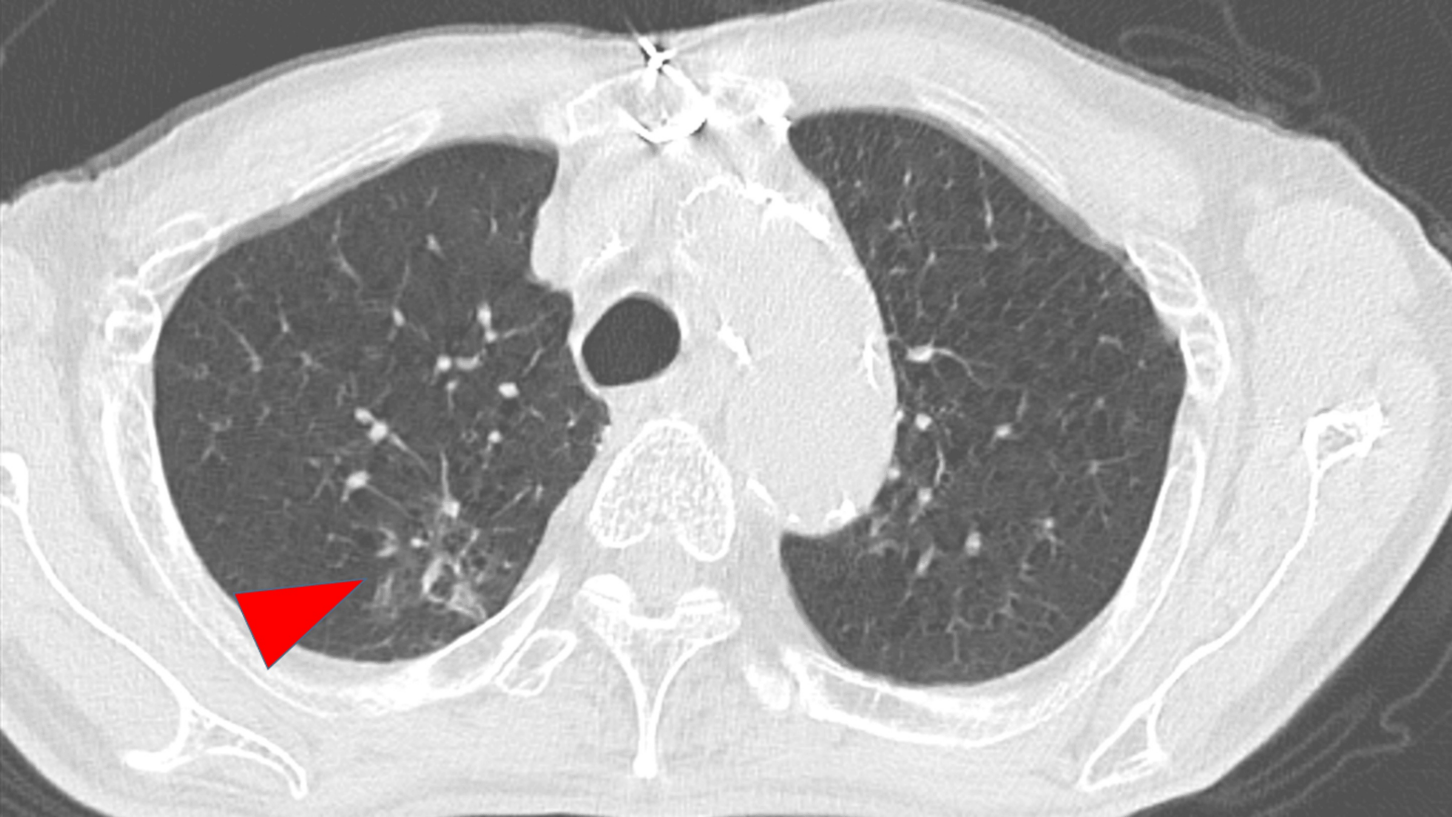
A lesion suggestive of lung cancer was observed in the right upper lobe on chest CT.

**Figure 3:**
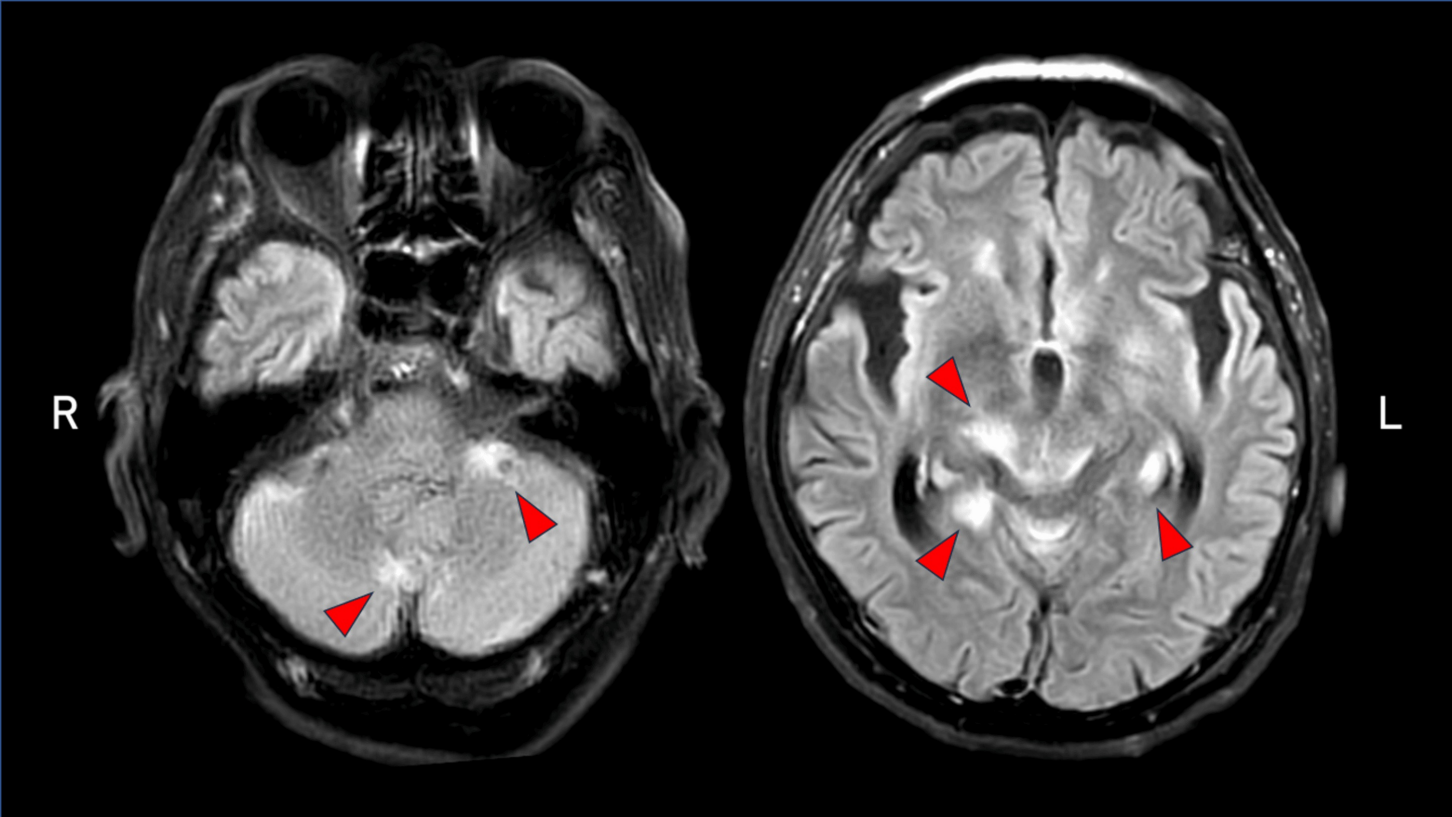
Fluid-attenuated inversion recovery axial MRI showing multiple high-intensity lesions in the cerebellum, periventricular, and hippocampus.

Blood tests and cerebrospinal fluid (CSF) examinations were conducted (Table 1). His inflammatory markers were elevated, and the CSF tested positive for VZV DNA by polymerase chain reaction. The result of blood culture was negative. Magnetic resonance imaging (MRI) showed multiple high intensities involving the cerebellum, periventricular cortex, and hippocampus (Figure 2), and the patient was diagnosed with meningoencephalitis due to disseminated herpes zoster. He was suspected to have lung cancer because of abnormal shadows on chest computed tomography (Figure 3), which led to testing for tumor markers (Table 1).

**Table 1:** Laboratory findings on admission Pro-GRP, pro-gastrin-releasing peptide; CYFLA, cytokeratin 19 fragment; mono, mononuclear cells; poly, polymorphonuclear cells

Test	Result	Normal Range
Hematological examinations		
White blood cell	10,740/μL	3,300-8,600/μL
C-reactive protein	5.08 mg/dL	0-0.14 mg/dL
Albumin	3.7 g/dL	4.1-5.1 g/dL
Glucose	108 mg/dL	70-109 mg/dL
Pro-GRP	334.6 pg/mL	0-81 pg/mL
CYFRA	20.6 ng/mL	0-3.5 ng/mL
Cerebrospinal fluid examinations		
Cell	314 cells/μL (poly. 40/μL, mono. 274/μL)	0-5/μL
Protein	1,363 mg/dL	10-40 mg/dL
Glucose	83 mg/dL	40-70 mg/dL

On the day of admission, we started treatment with intravenous acyclovir immediately, but the patient died of multiple organ failure on day 4 after admission.

## Discussion

This case involved an elderly male with VZV meningoencephalitis, who presented with acute progressive delirium. He was suspected to have lung cancer. These factors, older age and cancer, may have increased the risk of VZV reactivation due to an immunosuppressive state. He was misdiagnosed with Alzheimer’s disease since he had no skin eruptions in the early phases and his chief complaint was acute delirium.

Based on the loss of consciousness and the skin rash characteristic of VZV, we suspected VZV meningoencephalitis and were able to perform CSF testing and initiate treatment immediately.

On the day of admission to our hospital, we made the correct diagnosis and started treatment immediately; however, the prognosis was very poor. VZV meningoencephalitis is known to manifest without skin eruption [2,3]; however, as observed in this case, developing delirium in elderly patients makes proper diagnosis difficult.

VZV meningoencephalitis is believed to be caused by vasculitis due to direct infection of the cerebral vessels of VZV. Large vessel encephalitis with necrotizing or granulomatous vasculitis of large blood vessels is common in patients with normal immunity, whereas encephalitis with small artery lesions is common in immunocompromised patients [1]. The present patient was elderly and had possible lung cancer; therefore, it was thought to be a small vessel encephalitis caused by VZV.

## Conclusions

Immunocompromised patients may not present with fever in the early stages of meningoencephalitis. It is difficult to diagnose meningoencephalitis if the elderly develop delirium and do not have fever or skin rash. The possibility of meningoencephalitis should be kept in mind in elderly cancer patients presenting with cerebral small vessel lesions. Focusing on the generalized skin rash and performing a rapid antigen test for vesicles are important for the diagnosis of VZV meningoencephalitis.
